# Consensus and controversy in advanced urologic cancers: Insights from the Advanced Urologic Cancer Consensus Conference (AUC3) 2025

**DOI:** 10.3322/caac.70056

**Published:** 2025-12-13

**Authors:** Albert Jang, Yousef Zakharia, Pedro C. Barata

**Affiliations:** ^1^ Division of Medical Oncology, Department of Oncology Mayo Clinic Rochester Minnesota USA; ^2^ Division of Hematology and Medical Oncology, Department of Internal Medicine Mayo Clinic Phoenix Arizona USA; ^3^ Division of Solid Tumor Oncology, Department of Medicine University Hospitals, Cleveland Medical Center Case Western Reserve University School of Medicine Cleveland Ohio USA

Treatment advances in renal cell carcinoma (RCC) and urothelial carcinoma (UC) have redefined patient outcomes within a single decade, transforming fatal diseases into chronic, manageable conditions for many. The widespread adoption of multitargeted tyrosine kinase inhibitors (TKIs), immune checkpoint inhibitors (ICIs), and the antibody–drug conjugate enfortumab vedotin (EV) has extended survival compared with historic standards. Yet this progress has introduced new complexity, including overlapping mechanisms, inconsistent success across trials, and limited generalizability because of narrow eligibility criteria along with the lack of reliable predictive biomarkers.

To translate this rapidly expanding evidence base into clinical clarity, the Advanced Urologic Cancer Consensus Conference (AUC3) assembled more than 50 multidisciplinary experts from North America and Europe.^1^ By using a structured Delphi approach, the panel sought to define consensus thresholds and pinpoint gaps where data remain incomplete and controversial. This editorial highlights the most contentious issues (for which, even among global leaders, consensus was elusive) and frames future directions for investigation.

Pembrolizumab for high‐risk clear cell RCC (ccRCC) after nephrectomy remains the first adjuvant regimen to demonstrate an overall survival benefit compared with placebo, as demonstrated in KEYNOTE‐564 (ClinicalTrials.gov identifier NCT03142334).^2^ Strong consensus was reached (>90%) among the panel for high‐risk ccRCC including pathologic T3 (pT3) or worse and lymph node‐positive disease. Yet situations not addressed in the trial, such as stereotactic body radiation therapy to sites of oligometastatic disease within 1 year of nephrectomy, reached only 65.9% agreement for adjuvant pembrolizumab. Similarly, favorable pathology on the nephrectomy specimen (pT3a tumor, grade 1–2) reached only 54.5% in favor of adjuvant pembrolizumab, with the panel divided over treatment for lower risk patients.

Identifying low‐risk patients who may be spared the toxicity of ICI is critical, but predictive biomarkers are lacking. At 72 months, 48.7% of patients receiving placebo in KEYNOTE‐564 were still disease‐free compared with 58.5% of those receiving pembrolizumab,^3^ highlighting the importance of predicting the small subset of patients who truly derive benefit from pembrolizumab. Conversely, biomarkers of resistance are needed for the subset of patients likely to have disease relapse despite adjuvant pembrolizumab. They may require upfront adjuvant treatment intensification, and ongoing trials such as STRIKE of adjuvant pembrolizumab with tivozanib (ClinicalTrials.gov identifier NCT06661720) and the recently announced positive trial LITESPARK‐022^4^ of adjuvant pembrolizumab with belzutifan (ClinicalTrials.gov identifier NCT05239728) may offer insight.

Several approved frontline systemic therapies for metastatic ccRCC exist that can be grouped into ICI doublet or ICI‐TKI combinations. Consensus was strong (93.2%) for ipilimumab plus nivolumab with sarcomatoid differentiation, based on high response rates and longer progression‐free survival compared with sunitinib.^5^ In contrast, there was little consensus for rhabdoid differentiation, with 66.7% agreement to treat this similarly to sarcomatoid differentiation. For patients with International Metastatic RCC Database Consortium favorable‐risk disease, using an ICI doublet versus ICI‐TKI was evenly divided (50%) because the final long‐term follow‐up of CheckMate 214 (ClinicalTrials.gov identifier NCT02231749) demonstrated a more durable overall survival response for ipilimumab plus nivolumab over sunitinib.^6^ No consensus was reached for the optimal ICI‐TKI therapy given the lack of head‐to‐head comparisons. Indeed, long‐term outcomes for the various ICI‐TKI regimens appeared to be similar across the phase 3 trials when overall survival was stratified by International Metastatic RCC Database Consortium risk criteria.^7^

Clinical gaps remain in this frontline metastatic setting. Patients with brain metastases were excluded from these trials, so no consensus was reached on the optimal regimen. Oligometastatic disease remains controversial, including the definition of the number of metastases, with three or less sites the favored term (60.5%). The timing of oligometastatic recurrence when systemic therapy may be spared is uncertain. For a single site of metastasis developing within 6 months of nephrectomy, 39.5% opposed metastasis‐directed therapies because of the concern of aggressive disease biology requiring systemic therapy. Prospective studies for these cohorts of patients are required.

After frontline therapy, several approved options exist. Belzutifan is the latest approval after ICI and TKI progression; however, because of its novelty and unique mechanism of action as a hypoxia‐inducible factor 2 alpha inhibitor, there is uncertainty about using it early or after several lines of TKIs, with no consensus from the panel. Whether to challenge a patient’s refractory disease with another TKI versus belzutifan is unknown because the control arm in the LITESPARK‐005 trial (ClinicalTrials.gov identifier NCT04195750) was everolimus.^8^ Various hypoxia‐inducible factor 2 alpha inhibitors are being assessed in clinical trials in earlier treatment lines, which will likely cause even more confusion than clarity regarding the optimal regimens very soon.

The approval of adjuvant pembrolizumab also raises questions regarding ICI rechallenge with metastatic relapse. None of the published phase 3 trials accounted for an adjuvant ICI. Consensus was achieved by AUC3 to rechallenge with ICI 3–6 months after completion (78%) and beyond. However, these findings must be validated prospectively given negative ICI rechallenge in the metastatic setting based on the results of the CONTACT‐03 and TiNivo‐2 trials (ClinicalTrials.gov identifiers NCT04338269 and NCT04987203, respectively).^9^, ^10^

Although the abundant available evidence for ccRCC may lead to confusion over clarity, the lack of robust clinical trials for RCC of variant histologies means that there is no clarity. Other than collecting‐duct RCC and renal medullary carcinoma, no consensus was reached for the optimal regimen for each variant. Even for papillary RCC (the most common and best‐studied non‐ccRCC variant), only 68.3% favored ICI and TKI as the preferred regimen. Many variant RCC trials have historically enrolled several variant histologies simultaneously, given the low numbers of each individual subtype, while evaluating treatments directly extrapolated from ccRCC.^11^ Dedicated, subtype‐specific trials are urgently needed despite the low incidence of these variants because the outcomes of these variants are generally worse than those for ccRCC.

Whereas the treatment landscape for RCC is rich, albeit ambiguous, the UC field is navigating a different kind of complexity: how to integrate new agents into long‐standing platinum‐based backbones. For muscle‐invasive bladder cancer, the NIAGARA trial (ClinicalTrials.gov identifier NCT03732677) compared neoadjuvant gemcitabine, cisplatin, and perioperative durvalumab versus neoadjuvant gemcitabine plus cisplatin (GC).^12^ Therefore, it is unclear how this triplet from the NIAGARA trial would compare with neoadjuvant dose‐dense methotrexate, vinblastine, doxorubicin, and cisplatin, because the VESPER trial (ClinicalTrials.gov identifier NCT01812369) suggested that six cycles of dose‐dense methotrexate, vinblastine, doxorubicin, and cisplatin may yield better outcomes than four cycles of gemcitabine plus cisplatin.^13^ Consensus was not achieved, with the recommendation of the NIAGARA trial as the optimal neoadjuvant choice (66.7%). For the management of localized upper‐tract UC, there was no consensus given the limited efficacy for adjuvant nivolumab from a subset analysis of upper tract UC in CheckMate 274 (ClinicalTrials.gov identifier NCT02632409),^14^ whereas there are data for adjuvant chemotherapy in the POUT trial (ClinicalTrials.gov identifier NCT01993979).^15^

With enfortumab vedotin (EV) and pembrolizumab cemented as the optimal frontline systemic therapy regimen based on EV‐302,^16^ new issues have arisen. Although EV‐302 excluded patients with pre‐existing grade ≥2 peripheral neuropathy and uncontrolled diabetes mellitus, there was consensus to administer EV and pembrolizumab regardless in a real‐world setting. The optimal methods to manage EV side effects did not achieve consensus, including ways to control mild neuropathy and prolonged duration of skin rash. Adjuvant ICI has clouded the optimal time to initiate EV and pembrolizumab if metastatic disease develops, with just 38.9% agreeing that ≥6 months of prior ICI exposure to reintroduce an ICI.

After disease progression on EV and pembrolizumab, there is little consensus on the treatment besides the introduction of platinum‐based chemotherapy if a patient remains platinum‐eligible. The timing of trastuzumab deruxtecan (T‐DXd) if a tumor has HER2 immunohistochemical (IHC) 3+ (IHC3+) expression or erdafitinib for an *FGFR3* alteration remains to be clarified. For dual HER2 IHC3+ expression and an *FGFR3* alteration in the same tumor, the consensus was fully divided for using T‐DXd (38.9%) versus erdafitinib (30.6%) versus platinum chemotherapy (27.8%). These disagreements highlight the urgency for trials to evaluate the sequencing of regimens prospectively after EV and pembrolizumab. Further uncertainty regarding the management of frontline locally advanced and metastatic UC is anticipated in light of recent positive results from the KEYNOTE‐905/EV‐303 trial (ClinicalTrials.gov identifier NCT03924895), which investigated the combination of EV and pembrolizumab as neoadjuvant therapy for cisplatin‐ineligible patients who have muscle‐invasive bladder cancer or those who decline cisplatin chemotherapy.^17^

Like variant histologies of RCC, variant histologies of UC have been commonly overlooked in trials in all treatment settings.^18^ Treatment for predominant variants like squamous cell carcinoma of the bladder has generally been extrapolated from clinical trials focused on predominant urothelial histology. EV and pembrolizumab were favored over platinum‐based chemotherapy by the committee for predominant squamous histology, without reaching the consensus threshold (71.4% cisplatin‐ineligible, 68.6% cisplatin‐eligible). For pure squamous histology, consensus was achieved for platinum chemotherapy (75%) over EV and pembrolizumab (25%). Real‐world data suggest that outcomes for patients who have UC with histologic subtypes receiving EV are poor and potentially are even worse for those who have pure variant histologic subtypes.^19^, ^20^ The expression of nectin‐4 appears to be lower in those with variant histologies, which may be one component limiting the efficacy of EV.^21^ Hence, trials testing novel therapies dedicated to different subtypes of variant histologies, without combining the variants, are urgently needed.

The 2025 AUC3 consensus underscores both the remarkable progress and the persistent uncertainty across advanced RCC and UC. Shifts in expert opinion between pre‐meeting and post‐meeting rounds highlight the field's dynamic nature, in which new data continually reshape standards of care. The next wave of trials must prioritize biomarker‐driven selection, optimal sequencing of perioperative immunotherapy, and the integration of antibody–drug conjugates. As the AUC3 evolves annually, its iterative process will remain essential to transforming emerging evidence into actionable real‐world guidance for clinicians.

## CONFLICT OF INTEREST STATEMENT

Albert Jang reports personal fees from RealTimeCase; honoraria from MJH Life Sciences and Curio Science; and travel funds from MECC Inc. and the Binaytara Foundation outside the submitted work. Yousef Zakharia reports grants/research support from Eisai Inc., Exelixis, NewLink Genetics, and Pfizer; personal/consulting or advisory fees from AstraZeneca, Bristol Myers Squibb Company, Eisai Inc., EMD Serono, Exelixis, Genzyme Corporation, Gilead Sciences Inc., Janssen, Kyowa Kirin, Novartis, Pfizer, and SeaGen; and data safety and monitoring for Janssen Research and Development outside the submitted work. Pedro C. Barata reports grants or personal fees from Astellas; AstraZeneca; AVEO Oncology; Bayer; BMS; Dendreon; Eisai; EMD Serono; ESSA Pharma; Guardant Health; Ipsen; Caris Life Sciences; Exelixis; Janssen; Merck; Merus; MJH; Myovant; Novartis; Pfizer, Seattle Genetics; UroToday outside the submitted work.
